# Editorial: Malnutrition and Infections

**DOI:** 10.3389/fnut.2022.897780

**Published:** 2022-04-06

**Authors:** Julio Villena, Haruki Kitazawa, Imad Al Kassaa, Susana Alvarez

**Affiliations:** ^1^Laboratory of Immunobiotechnology, Reference Centre for Lactobacilli (CERELA-National Council of Scientific and Technological Research), San Miguel de Tucuman, Argentina; ^2^Food and Feed Immunology Group, Laboratory of Animal Products Chemistry, Graduate School of Agricultural Science, Tohoku University, Sendai, Japan; ^3^Livestock Immunology Unit, International Education and Research Center for Food and Agricultural Immunology (CFAI), Graduate School of Agricultural Science, Tohoku University, Sendai, Japan; ^4^Faculty of Public Health, Lebanese University, Beirut, Lebanon

**Keywords:** malnutrition, infections, immunology, beneficial microbes, metabolism and immunity

Food microorganisms and macro- and micro-nutrients play pleiotropic effects on the host health. These microbes and molecules are involved in the normal function and modulation of all the biological processes, including the maintenance of healthy mucosal barriers and immune responses that protects the host against invading pathogens. Scientific advances have revealed how microorganisms and nutrients influence the resistance to infections. In this Research Topic Scientific papers are gathered that are in line with this knowledge, and that contribute to the advancement of this discipline.

The impact of food microorganisms in the local (intestinal) mucosa has been well-characterized in the past decades. In this regard, the study conducted by Argentinian and Japanese laboratories in collaboration demonstrated that the use of *Ligilactobacillus salivarius* strains isolated from wakeame-fed pigs may help to increase the resistance to rotavirus infection as well as to the superinfection caused by rotavirus and enterotoxigenic *Escherichia coli* (Indo et al.). In particular, *L. salivarius* FFIG58 was shown to modulate the production of IFN-γ and IFN-β thought the regulation of TLR3 signaling in the intestinal mucosa. The FFIG58 also increased the resistance to the intestinal pathogens in a mice model.

Interestingly, it was shown that food microorganisms and nutrients can also influence the immune responses and the resistance to infections in distal sites from the gastrointestinal tract such as the skin or the mammary or respiratory mucosa. The opinion article of Saini and Rai highlights that malnutrition affects visceral leishmaniasis and its dermal complications because of the reduced immune response and increased visceralization of the parasite in malnourished hosts. In particular, authors emphasized the potential role of linoleic acid deficiency in the skin complications of the visceral leishmaniasis patients and suggest that dietary supplementation with linoleic acid may help to improve immune responses against *Leishmania* infection. On the other hand, *Streptococcus uberis* is able to cause a critical mammary gland infection in the bovine host by triggering inflammation and metabolic disorders. This metabolic disorder is characterized by the up-regulation of glycolysis and oxidative phosphorylation in mammary epithelial cells. Lan et al., demonstrated that the pretreatment of mice with taurine before *S. uberis* infection restores metabolic homeostasis, reverses metabolic dysfunction by decrease of lipid, amino acid and especially energy disturbance in the infectious context. In addition, the work reported that taurine help to alleviate the excessive inflammatory response and reduce cellular damage through the activation of the AMPK–mTOR pathway.

In this Research Topic, three articles highlights the importance of food nutrients and microorganisms in the resistance of the host to respiratory infections. In the review of Salva et al., the authors described the impact of protein malnutrition on the respiratory immune system. The article also described the effectiveness of immunomodulatory lactic acid bacteria used as supplements in repletion diets to restore the normal function of the respiratory immune system and to increase the resistance against *Streptococcus pneumoniae* infection in recovered malnourished hosts. Particular attention is given to the alterations induced by protein deficiency in the development of phagocytic cells, T and B lymphocytes in bone marrow, as well as the effectiveness of the probiotic strain *Lacticaseibacillus rhamnosus* CRL1505 or its peptidoglycan to improve the recovery of hematopoiesis and the respiratory immune system. The second article describes that stroke-associated pneumonia is risk in acute ischemic stroke patients that is caused by the interruption of blood flow to specific areas of the brain (Yan et al.). The work showed that a high lactic dehydrogenase to albumin ratio might be a potential predictor for the incidence of stroke-associated pneumonia. Authors also suggested that the low albumin levels could be associated to poor nutritional reserves and thus, to a weak immune system, which may increase the risk of pneumonia. The third article describes that patients with cystic fibrosis are highly susceptible to respiratory infections because of their progressive lung failure (Sealy et al.). These patients have recurrent infections with bacteria and also with virus that damage the respiratory mucosa and enhance patient's susceptibility to bacterial infections. Interestingly, the work suggested that the monitoring of vitamin A level in cystic fibrosis patients could be crucial to decrease the impact of lung infection and reduce lung lesions. In addition, by using a cystic fibrosis mouse model, the authors demonstrated that oral vitamin A supplements significantly reduce lung lesions that would otherwise persist for 5–6 weeks post-virus exposure.

The original research articles, opinion and review of this Research Topic provide a comprehensive set of information on the influence of food microorganisms and nutrients on the resistance to infections through the modulation of the immune system. The editors hope that this Research Topic will serve as a stimulus to improve the research in this exciting area of the Nutritional Immunology.

## Author Contributions

All authors listed have made a substantial, direct, and intellectual contribution to the work and approved it for publication.

## Conflict of Interest

The authors declare that the research was conducted in the absence of any commercial or financial relationships that could be construed as a potential conflict of interest.

## Publisher's Note

All claims expressed in this article are solely those of the authors and do not necessarily represent those of their affiliated organizations, or those of the publisher, the editors and the reviewers. Any product that may be evaluated in this article, or claim that may be made by its manufacturer, is not guaranteed or endorsed by the publisher.

